# A national intervention to increase representation of Latinos/Hispanics in clinical trials on dementia: A mixed‐method evaluation

**DOI:** 10.1002/alz.090985

**Published:** 2025-01-09

**Authors:** Adriana Perez, Elena Portacolone, Maria P. Aranda

**Affiliations:** ^1^ University of Pennsylvania, Philadelphia, PA USA; ^2^ Leonard Davis Institute of Health Economics, PHILADELPHIA, PA USA; ^3^ University of California San Francisco, San Francisco, CA USA; ^4^ Philip Lee Institute on Health Policy Studies, San Francisco, CA USA; ^5^ University of Southern California, Los Angeles, CA USA; ^6^ USC Edward R. Roybal Institute on Aging, University of Southern California, Los Angeles, CA USA

## Abstract

**Background:**

The lack of inclusion of Latinos/Hispanics in Alzheimer’s disease and related dementias (ADRD) clinical trials reduces the generalizability of study findings and hinders our understanding of the mechanisms of dementia, further widening cognitive health disparities. To address this growing public health concern, the purpose of this study is to leverage the national infrastructure of a Consortium between the National Association of Hispanic Nurses (NAHN) and the Alzheimer’s Association (ALZ) to increase the representation Latino/Hispanic participants in one clinical trial on dementia.

**Methods:**

The study used a randomized controlled trial design and mixed‐method evaluation. Recruitment intervention strategies were tested across 4 cities with high Latino/Hispanic populations. Metrics of sites receiving the intervention vs. those using usual recruitment activity in a clinical trial on dementia. Focus groups and semi‐structured interviews captured the experiences and perceptions of recruitment procedures from the research team, officers of NAHN and ALZ and potential participants who chose to enroll in the clinical trial.

**Results:**

Across the 4 intervention sites, a total of 145 Latino potential participants were referred to the clinical trial; however, none were enrolled. Themes that emerged from focus groups and interviews include: 1) Thirst for information about dementia in Latino communities; 2) A sense of being stronger and bigger together stemming from the partnership between NAHN and ALZ; 3) Importance of logistical support offered by the research team; 4) Importance of timely and thorough follow up with Latino adults who expressed intention to participate in the clinical trial.

**Conclusion:**

Study findings underline the importance of focusing on structural mechanisms enhancing Latino representation. Findings can inform future recruitment science efforts among researchers, community leaders, and policymakers to capture and promote the beneficial roles of collaborations between academia and national organizations that are embedded within communities historically underrepresented in ADRD clinical trials.

